# Supplementary material for: The effect of population-based blood pressure screening on long-term cardiometabolic morbidity and mortality in Germany: A regression discontinuity analysis
Source: PLoS Med. 2022 Dec 27;19(12):e1004151. doi: 10.1371/journal.pmed.1004151 (PMC9848470; doi:10.1371/journal.pmed.1004151)
Supplement: S1 Appendix — (PDF) [file pmed.1004151.s001.pdf]

S1 Appendix: Collection of drug information

Intake of antihypertensive medications in the last seven days was measured by a computer-assisted drug recording procedure, which included both drug package collection and self-reported information. Antihypertensive drugs were classified as such following recommendations of the German Hypertension League (Deutsche Hochdruckliga) in the year of the respective survey. For example, for the study S4, following recommendations were followed:

Empfehlungen zur Hochdruckbehandlung (Recommendation for the treatment of hypertension). 15th ed. Heidelberg: Deutsche Liga zur Bekämpfung des hohen Blutdruckes e.V.; November 1999.
