# Supplementary material for: The effect of population-based blood pressure screening on long-term cardiometabolic morbidity and mortality in Germany: A regression discontinuity analysis
Source: PLoS Med. 2022 Dec 27;19(12):e1004151. doi: 10.1371/journal.pmed.1004151 (PMC9848470; doi:10.1371/journal.pmed.1004151)
Supplement: S2 Appendix — (PDF) [file pmed.1004151.s002.pdf]

S2 Appendix: Imputation information

Our initial sample contained missing values for several variables that are relevant for our study. S2 Table provides an overview of the number of missing values. Instead of dismissing incomplete cases from our analytic sample, we decided to increase the statistical power of our analysis by imputing missing values. We applied a predictive mean modelling (pmm) multiple imputation approach to the initial sample, using the MICE package in R.

In this step, we also imputed observations in the outcome variables: We further imputed 102 missing observations for the death variable (0.6%), 345 missings in the MI incidence variable (2.0%) and 987 missings in the stroke incidence variable (5.6%) (see S2 Table).

After imputing missing values, some inconsistencies regarding the time variables appeared. In case of 150 observations, either the imputed myocardial infarction (MI) or the stroke incidence time variable were larger than the variable that indicated the time of death / the end of the observation period. Since these imputed values are unrealistic, we decided to adjust the respective time variables to the time of death / the end of the observation period.

S2 Table summarizes our data before and after the imputation[1]. The imputation did not change the characteristics of the sample substantially. Nevertheless, to make sure that the imputation did not bias our results, we also ran the primary outcome analyses of this study only considering complete cases as a robustness check (S6 Figure).

[1] Please note that for the main analysis the observation period was cut to 16.9 years after imputation. Hence, the numbers regarding CVD event indicators vary substantially from the numbers presented in Table 1 of the main paper.
