# Supplementary material for: The effect of population-based blood pressure screening on long-term cardiometabolic morbidity and mortality in Germany: A regression discontinuity analysis
Source: PLoS Med. 2022 Dec 27;19(12):e1004151. doi: 10.1371/journal.pmed.1004151 (PMC9848470; doi:10.1371/journal.pmed.1004151)
Supplement: S3 Appendix — (PDF) [file pmed.1004151.s003.pdf]

S3 Appendix: Supplementary information on the intervention

The analyzed intervention was carried out via mail, precisely by communicating the results of a comprehensive screening in the included studies via a results letter. Individuals received a personalized feedback based on the results of the blood pressure measurements carried out by trained personnel in the study center:

- Individuals with a blood pressure < 140/90 mmHg received the following message:

  "*According to the recommendations of the World Health Organization (WHO), values below 140 / 90 mmHg are considered normal. Your blood pressure lies in this range."*
  [Original: Die Weltgesundheitsorganisation (WHO) bezeichnet Blutdruckwerte bis 139 mmHg systolisch und 89 mmHg diastolisch als normal. Ihr Wert lag in diesem Bereich."]

- Individuals with a blood pressure ≥ 140/90 mmHg - < 160/95 mmHg received the following message:

  "*The World Health Organization (WHO) defines blood pressure values from 140 to below 160 mmHg systolic and/or from 90 to below 95 mmHg diastolic as requiring control. Your value was in this range. Please have your blood pressure checked by your general practitioner, when you have the opportunity*."

  ["Die Weltgesundheitsorganisation (WHO) bezeichnet Blutdruckwerte von 140 bis 159 mmHg systolisch und/oder 90 bis 94 mmHg diastolisch als kontrollbedürftig. Ihr Wert lag in diesem Bereich. Bitte lassen Sie Ihren Blutdruck gelegentlich durch Ihren Hausarzt überprüfen."]

Notes:

- Individuals with a blood pressure ≥ 160/95 mmHg received the following message: "*The World Health Organization (WHO) defines blood pressure values of 160 mmHg systolic and/or 95 mmHg diastolic as hypertension (high blood pressure). Your value was within this range. Please have your blood pressure checked by your general practitioner soon*." Due to a lack of power, we were not able to investigate the impact of this threshold.
- In the S4 study, the communicated values were an average between the 2nd and 3rd blood pressure measurement. In the S1-S3 studies, the communicated values were based exclusively on the 3rd measurement.
- In the S4 study, the words "when you have the possibility" ("gelegentlich") were omitted from the personalized communication.
- On the same results letter, individuals received a similar personalized feedback on several other aspects. All letters included information on blood pressure, blood analyses (cholesterol, uric acid, and creatinine). Depending on the survey, participants received also information on further parameters (Gamma-GT, serum glucose, HbA1c, Erythrocytes, Hemoglobin, Hematocrit, Thrombocytes, Leukocytes, MCV, MCH), results of an OGTT test, electrocardiography, urinalysis, body composition analysis, non-mydriatic analysis of the eye, height and weight.
