# Supplementary figures and images for: The effect of population-based blood pressure screening on long-term cardiometabolic morbidity and mortality in Germany: A regression discontinuity analysis
Source: PLoS Med. 2022 Dec 27;19(12):e1004151. doi: 10.1371/journal.pmed.1004151 (PMC9848470; doi:10.1371/journal.pmed.1004151)

**S1 Figure: Flowchart of the sample construction for the main analysis**

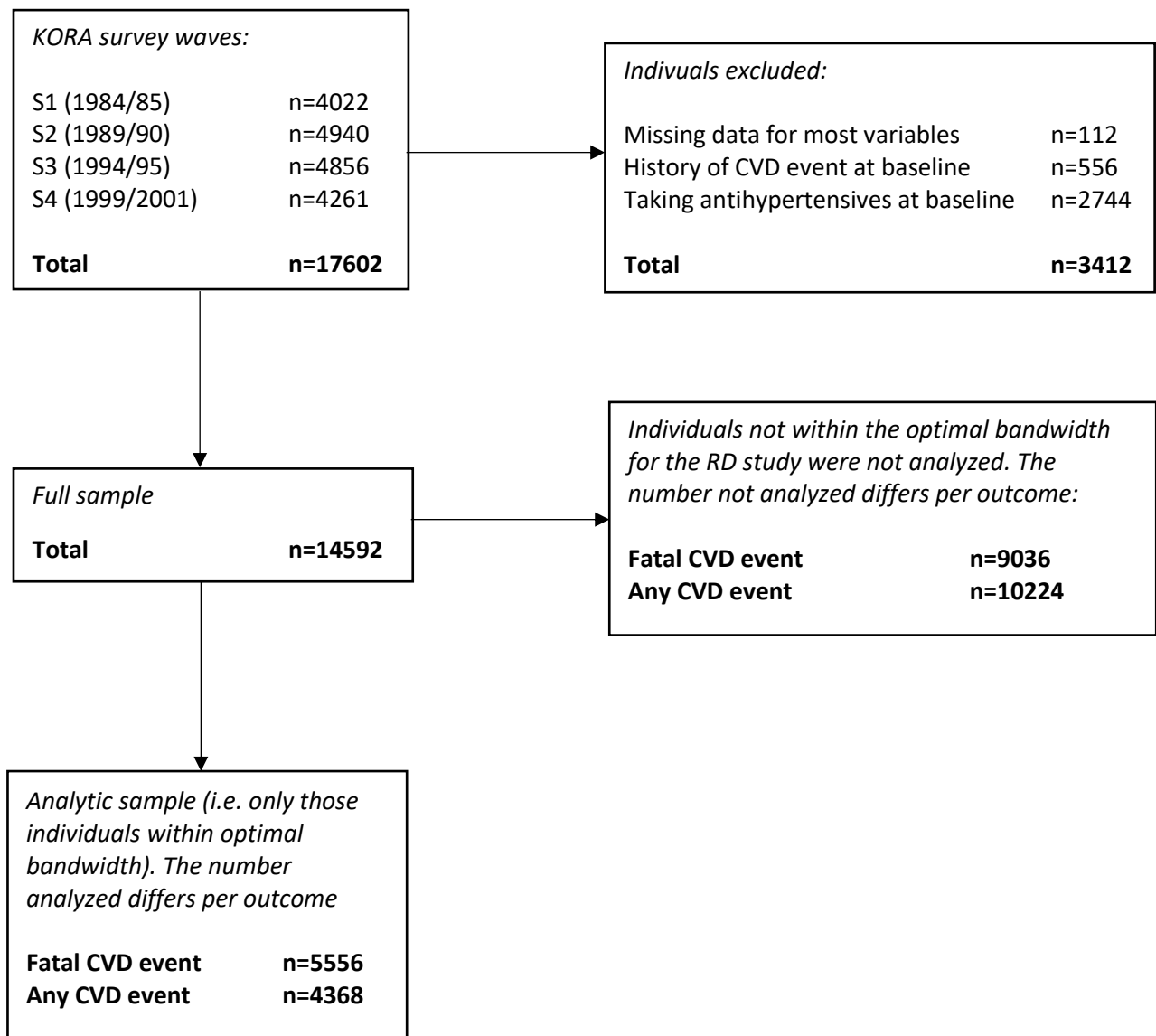

Supplement: S1 Fig — (PDF) [file pmed.1004151.s005.pdf]

**S2 Figure: Flowchart of the sample construction for the secondary outcomes analysis**

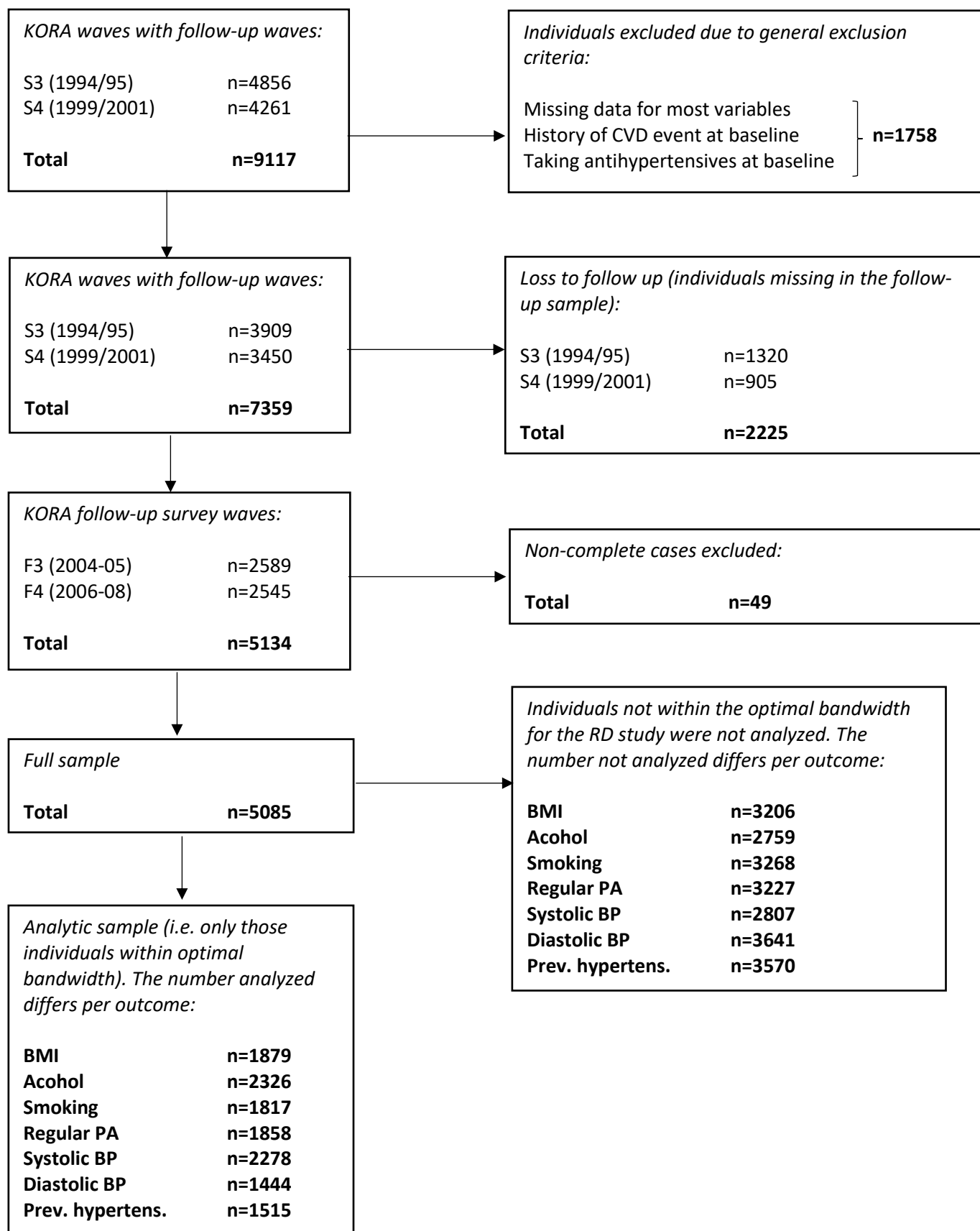

Supplement: S2 Fig — (PDF) [file pmed.1004151.s006.pdf]

**S3 Figure: Density check for the intermediate outcomes sample (at baseline)**

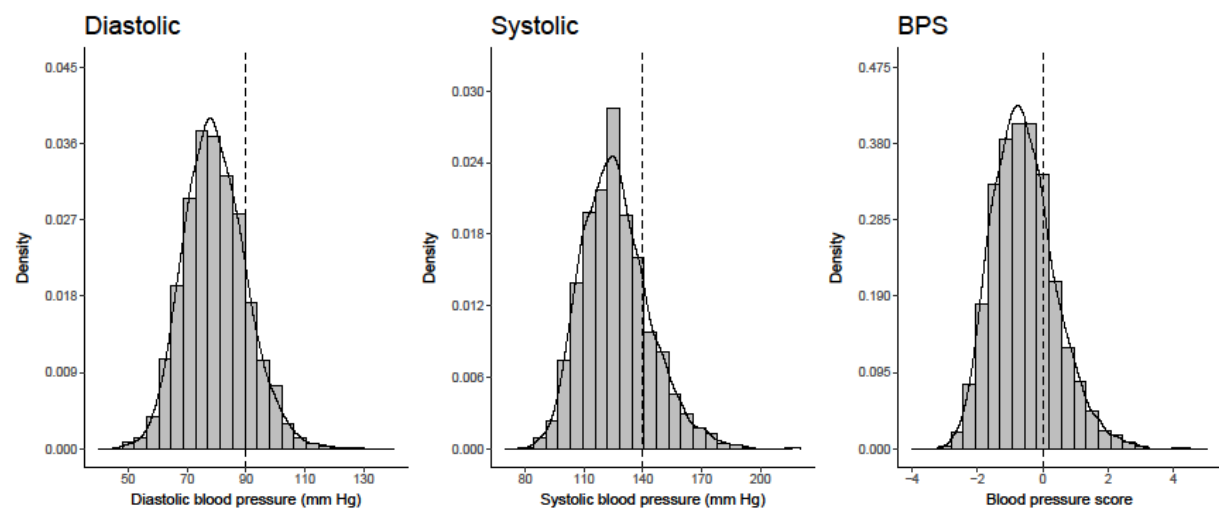

Supplement: S3 Fig — (PDF) [file pmed.1004151.s007.pdf]

**S4 Figure: Continuity of covariates (at baseline)**

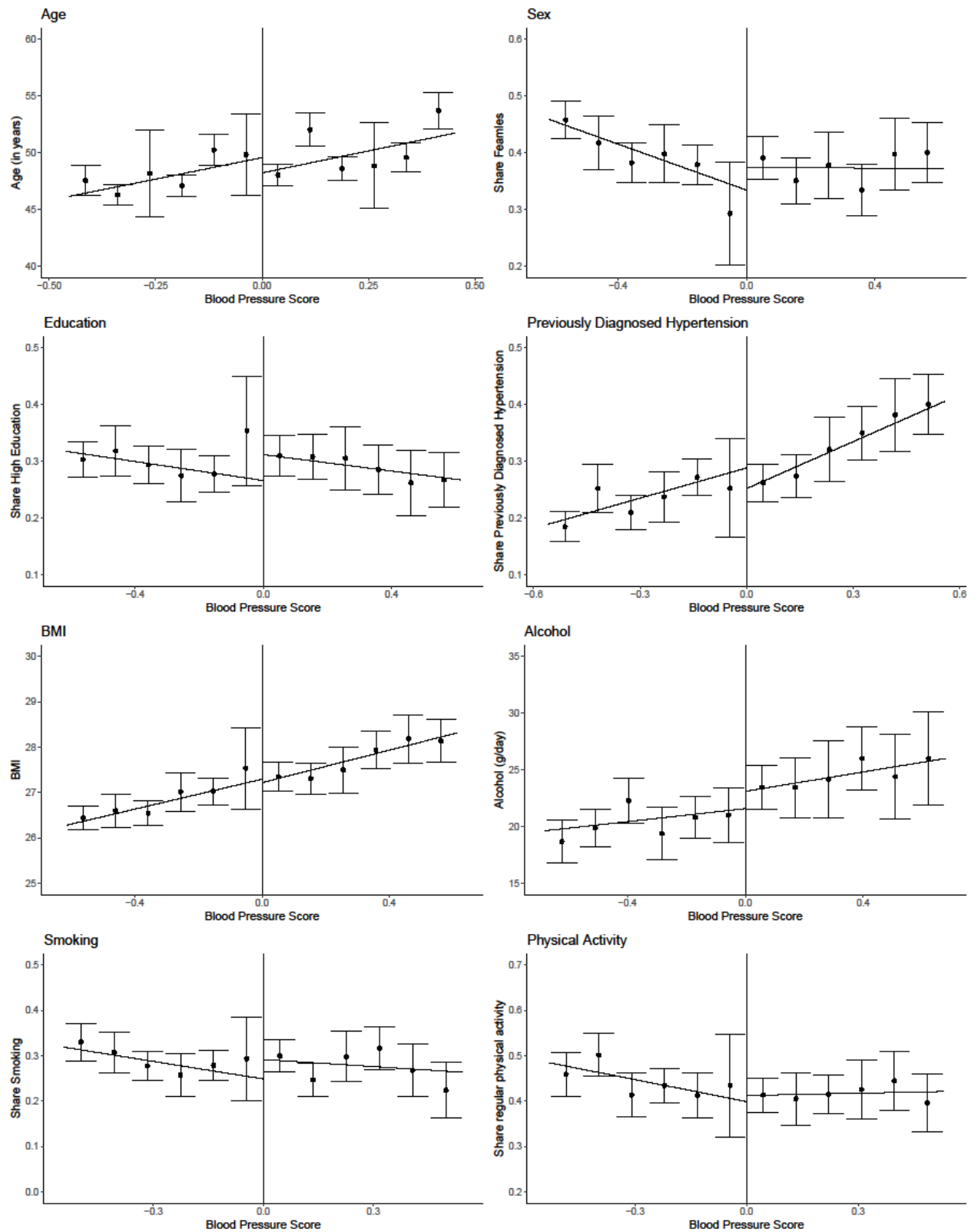

Supplement: S4 Fig — (PDF) [file pmed.1004151.s008.pdf]

**S5 Figure: Graphical representation of the effect at the threshold for different bandwidths**

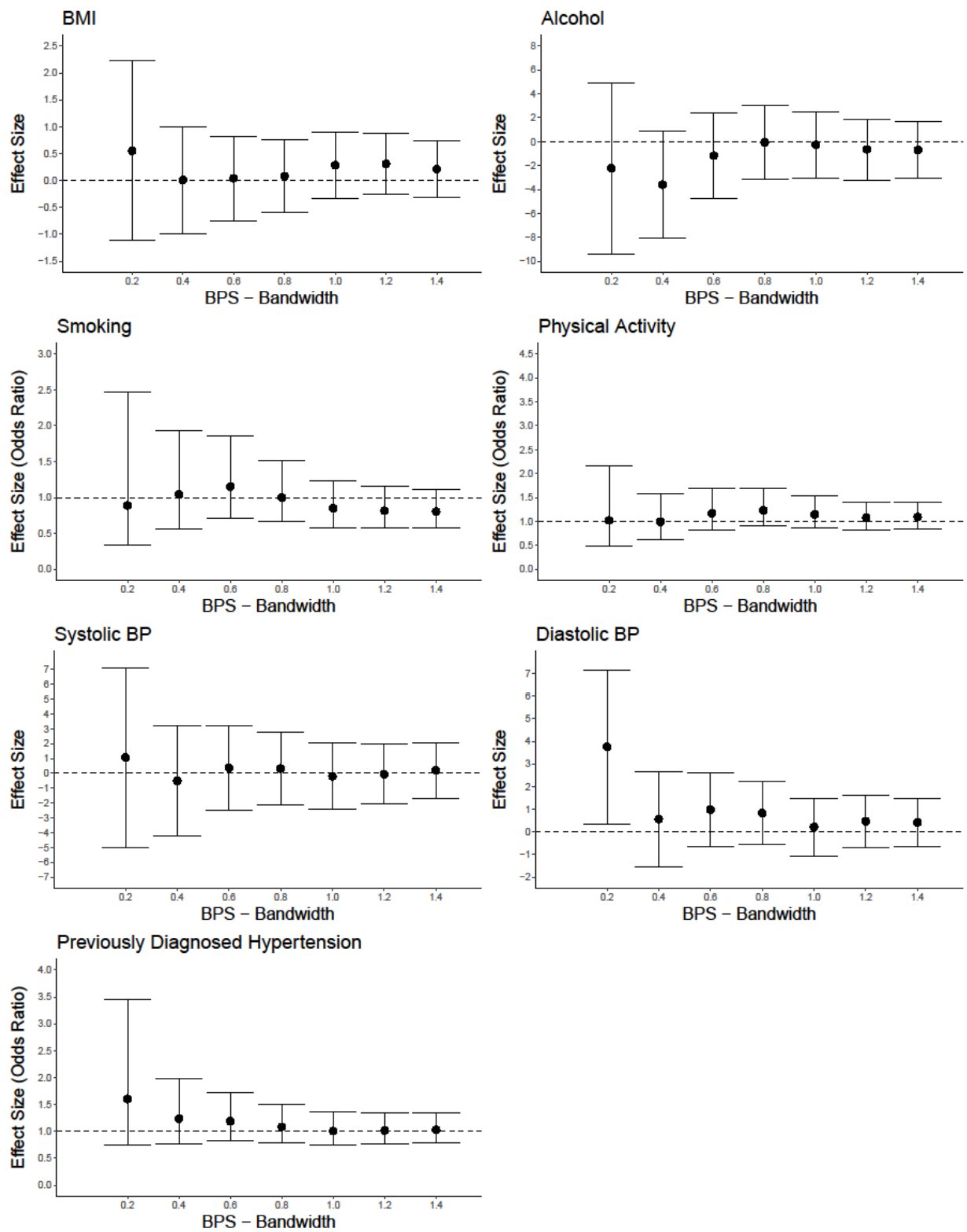

Supplement: S5 Fig — (PDF) [file pmed.1004151.s009.pdf]

**S6 Figure: Graphical representation of the analysis results with original data**

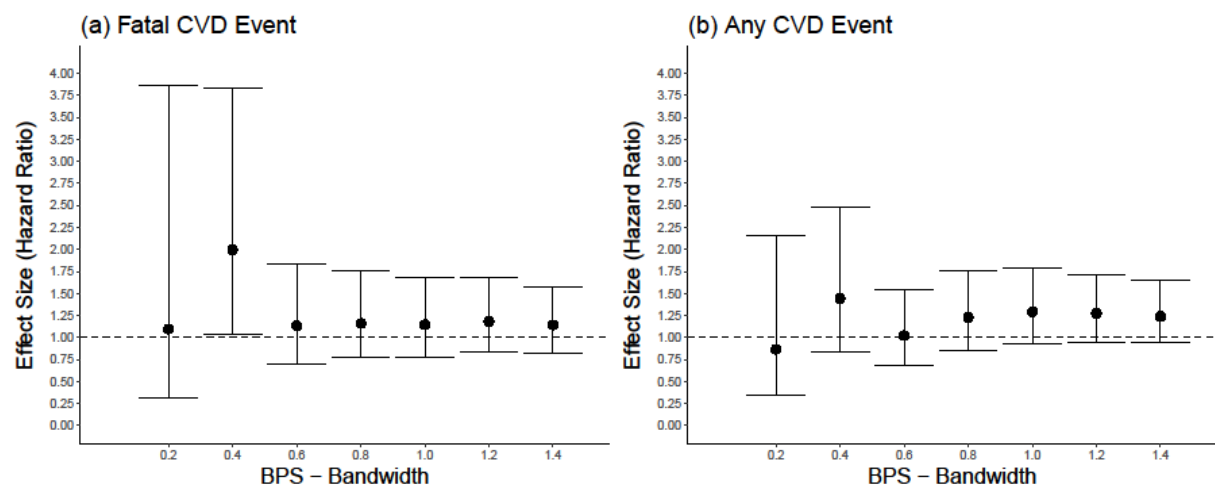

Supplement: S6 Fig — (PDF) [file pmed.1004151.s010.pdf]
