# Supplementary material for: The effect of population-based blood pressure screening on long-term cardiometabolic morbidity and mortality in Germany: A regression discontinuity analysis
Source: PLoS Med. 2022 Dec 27;19(12):e1004151. doi: 10.1371/journal.pmed.1004151 (PMC9848470; doi:10.1371/journal.pmed.1004151)
Supplement: S1 Table — (PDF) [file pmed.1004151.s011.pdf]

S1 Table: Full descriptive statistics

Variables	Sample Selection		Sample within optimal BW*		Secondary Outcomes Sample**	
	Initial	Final	Fatal CVD Event	Any CVD Event	Baseline	Follow up
N	17490	14592	5556	4368	5085	5085
General Characteristics						
Age (Mean)	48.65	46.18	48.24	48.52	46.13	-
Female (%)	50.13	50.04	39.2	37.64	51.31	-
High Education (%)	29.62	31.3	29.12	29.49	38.8	-
BMI (Mean)	26.85	26.33	27.11	27.16	26.42	27.2
Alcohol (Mean g/day)	18.6	19.35	22.21	22.49	16.49	15.2
Smoking (%)	26.8	29.18	28.55	28.32	25.39	20.04
Regular Physical Activity (%)	42.27	44.77	43.36	42.79	51.5	55.32
Systolic BP (Mean mm Hg)	130.56	128.18	134.61	135.33	126.39	125.09
Diastolic BP (Mean mm Hg)	80.18	79.67	84.01	84.21	79.58	78.51
Previously Diagnosed Hypertension	32.22	22.15	26.57	27.08	21.75	39.69
KORA Study (%)						
S1 (1984/85)	21.3	23.05	23.9	23.28	-	-
S2 (1989/90)	27.02	26.52	28.17	28.34	-	-
S3 (1994/95)	27.38	26.79	27.77	26.97	50.3	-
S4 (1999/2001)	24.31	23.64	20.16	21.41	49.7	-
Exclusion Criteria (%)						
Antihypertensive Med. Intake	15.69	0	0	0	0	20.16
Previous MI	1.97	0	0	0	0	-
Previous Stroke	1.21	0	0	0	0	-
Primary Outcomes (%)						
Fatal CVD Event	7.19	4.37	4.95	5.22	1.95	-
Any CVD Event	9.37	6.02	6.61	6.98	4.52	-

Notes: BW=bandwidth, CVD=cardiovascular disease, BMI=body mass index, BP=blood pressure, Med.=Medication, MI=myocardial infarction; mm HG=millimeters mercury
* the optimal BW of the primary outcomes.
** full secondary outcome sample (no bandwidth restriction).
