# Supplementary material for: The effect of population-based blood pressure screening on long-term cardiometabolic morbidity and mortality in Germany: A regression discontinuity analysis
Source: PLoS Med. 2022 Dec 27;19(12):e1004151. doi: 10.1371/journal.pmed.1004151 (PMC9848470; doi:10.1371/journal.pmed.1004151)
Supplement: S2 Table — (PDF) [file pmed.1004151.s012.pdf]

S2 Table: Imputation data

Variables	Missings (N)	Before Imputation (%)	After Imputation (%)	After - Before
N		17490	17490	
General Characteristics				
Age (Mean)	0	48.65	48.65	0
Female (%)	0	50.13	50.13	0
High Education (%)	15	29.63	29.62	-0.01
BMI (Mean)	222	26.85	26.85	0
Alcohol (Mean g/day)	31	18.59	18.6	0.01
Smoking (%)	6	26.81	26.8	-0.01
Regular Physical Activity (%)	32	42.29	42.27	-0.02
Systolic BP (Mean mm Hg)	18	130.57	130.56	-0.01
Diastolic BP (Mean mm Hg)	18	80.18	80.18	0
Previously diagnosed hypertension (%)	13	32.22	32.22	0
KORA Study (%)				
S1 (1984/85)	0	21.3	21.3	0
S2 (1989/90)	0	27.02	27.02	0
S3 (1994/95)	0	27.38	27.38	0
S4 (1999/2001)	0	24.31	24.31	0
Exclusion Criteria (%)				
Antihypertensiva Intake	15	15.69	15.69	0
Previous MI	0	1.97	1.97	0
Previous Stroke	0	1.21	1.21	0
Event Indicators (%)				
Death	0	28.73	28.73	0
CVD Death	102	5.98	5.96	-0.02
CHD Death	102	12.2	12.22	0.02
Stroke Death	102	2.12	2.13	0.01
MI Incidence	345	9.48	10.04	0.56
Stroke Incidence	987	9.48	9.05	-0.43
Time Variables (in days)				
Death	0	7420.94	7420.94	0
MI	345	7044.16	6991.24	-52.92
Stroke	987	6594.49	6492.47	-102.02

Notes:BP: blood pressure; CHD: Coronary heart disease; CVD: Cardiovascular disease; MI: Myocardial infarction;
