# Supplementary material for: The effect of population-based blood pressure screening on long-term cardiometabolic morbidity and mortality in Germany: A regression discontinuity analysis
Source: PLoS Med. 2022 Dec 27;19(12):e1004151. doi: 10.1371/journal.pmed.1004151 (PMC9848470; doi:10.1371/journal.pmed.1004151)
Supplement: S3 Table — (PDF) [file pmed.1004151.s013.pdf]

S3 Table: Description of analytic sample for the outcome "any CVD event"

General characteristics	Full sample	Analytic sample* Any CVD Event: Sample	Below cutoff	Above cutoff
N	14,592	4,368	2,296	2,072
Age (years, SD)	46 (13)	49 (13)	48 (13)	50 (12)
Female	50%	38%	38%	37%
High education	31%	29%	29%	30%
BMI (kg/m^2, SD)	26 (4)	27 (4)	27 (4)	28 (4)
Alcohol (g/day, SD)	19 (26)	22 (28)	21 (26)	24 (29)
Smoking	29%	28%	28%	29%
Regular physical activity	45%	43%	44%	42%
Previously diagnosed hypertension	22%	27%	24%	30%
Systolic BP (mmHg, SD)	128 (18)	135 (7)	132 (6)	139 (7)
Diastolic BP (mmHg, SD)	80 (11)	84 (7)	82 (6)	87 (7)
N events		305 (7%)	131 (6%)	174 (8%)

Notes: CVD: cardiovascular disease, SD: standard deviation; BP: blood pressure, CI: confidence interval;
* within the respective optimal bandwidths.
