# Supplementary material for: The effect of population-based blood pressure screening on long-term cardiometabolic morbidity and mortality in Germany: A regression discontinuity analysis
Source: PLoS Med. 2022 Dec 27;19(12):e1004151. doi: 10.1371/journal.pmed.1004151 (PMC9848470; doi:10.1371/journal.pmed.1004151)
Supplement: S4 Table — (PDF) [file pmed.1004151.s014.pdf]

**S4 Table: Full information on covariate balance tests**

| | Opt. BW | N | Model | beta | OR | 95%-CI |
|---|---|---|---|---|---|---|
| Age | 0.471 | 4368 | Linear | -1.331 | - | [-2.877, 0.215] |
| Female | 0.621 | 5646 | Logit | 0.165 | 1.179 | [0.952, 1.464] |
| High Education | 0.622 | 5646 | Logit | 0.22 | 1.246 | [0.991, 1.568] |
| Previously diagnosed hypertension | 0.561 | 5556 | Logit | -0.195 | 0.823 | [0.648, 1.047] |
| BMI (kg/m$^2$) | 0.631 | 5646 | Linear | -0.075 | - | [-0.495, 0.345] |
| Alcohol (Mean g/day) | 0.681 | 6277 | Linear | 1.524 | - | [-1.134, 4.182] |
| Smoking | 0.544 | 5060 | Logit | 0.211 | 1.235 | [0.969, 1.575] |
| Regular Physical Activity | 0.536 | 4491 | Logit | 0.054 | 1.055 | [0.829, 1.344] |

**Notes:** BW=optimal bandwidth; beta=result from the respective model; OR=Odds-ratio; CI=confidence interval.
