# Supplementary material for: The effect of population-based blood pressure screening on long-term cardiometabolic morbidity and mortality in Germany: A regression discontinuity analysis
Source: PLoS Med. 2022 Dec 27;19(12):e1004151. doi: 10.1371/journal.pmed.1004151 (PMC9848470; doi:10.1371/journal.pmed.1004151)
Supplement: Attachment — Submitted filename: pedron.pdf [file pmed.1004151.s015.pdf]

This paper uses a cohort of people involved in blood pressure screening studies in a city in southern Germany and uses regression discontinuity technology to examine cardiovascular outcomes.

I was asked for a statistical report and I interpret that to include all aspects of the design and conduct of the study.

Points of detail

Page 1 I wonder whether the title should include the location? I suggest Germany rather than Bavaria or Augsburg.

Page 6 and elsewhere The phrase 'information treatment' does not seem quite right to me as it made me think that it was the information which was being treated. How about 'information intervention'?

Page 7 I think including the question in the original language is a good idea since the authors' gloss clarifies an important point that diagnosed here did not mean by a registered medical practitioner which I suspect many Anglophones would have assumed..

Page 9 Using μ for the error seems unusual, I would have expected ϵ. For completeness defining β_i would be good.

Page 12 It might be best to make clear how the density plots demonstrate the absence of manipulation

Page 14 I think strictly speaking the analysis failed to find an effect rather than indicating no effect. A similar phrase is found on page 13.

Page 18 Although the regression discontinuity design cannot be used to examine what happens to people with much higher blood pressures it seems a shame not to know anything about them. Perhaps this is better treated in a separate paper.

Page 20 Claiming to be the first is rather bold. First to our knowledge is safer.

Points of more substance

The authors have apparently truncated problematic times in their imputation. I cannot pretend to be the world's leading expert on `mice` but I think the facility to do passive imputation would have solved the problem of the impossible times in a more principled way. One of the package vignettes

appears to have an example of imposing a similar constraint. These are hard to find, they are linked from page 78 in the manual on CRAN (`mice` version 3.14.0).

It may be that it is in fact impossible to do as I suggest and it is, of course, reassuring that the complete cases analysis is not substantively different.

Incidentally I think providing a citation for `mice` would be a nice idea. Package authors are working for free and this may be the only credit they get. (Full disclosure: I am a CRAN author although I have no connection with `mice`.)

Summary

Mostly for improvement or clarification.

Michael Dewey
