# Supplementary material for: The effect of population-based blood pressure screening on long-term cardiometabolic morbidity and mortality in Germany: A regression discontinuity analysis
Source: PLoS Med. 2022 Dec 27;19(12):e1004151. doi: 10.1371/journal.pmed.1004151 (PMC9848470; doi:10.1371/journal.pmed.1004151)
Supplement: Attachment — Submitted filename: Response to reviewers.docx [file pmed.1004151.s016.docx]

**Response to reviewers – 1st revision round – PLOS medicine**

**Pedron et al.**

We thank the reviewers and the editors for their very useful comments and improvement suggestions. Below you find our point-to-point answer.

**Editor´s comments**

1. **Please include the setting in your title (i.e., “The effect of population-based blood pressure screening on long-term cardio-metabolic morbidity and mortality in Germany: A regression discontinuity analysis”).**

We included the location in the title. The new title is now:

*“The effect of population-based blood pressure screening on long-term cardio-metabolic morbidity and mortality in Germany: A regression discontinuity analysis”*

2. **Please add this statement to the manuscript's Competing Interests: "****TB is editor-in-chief of PLOS Medicine.”**

We added this piece of information in the competing interest statement.

3. **Please structure your abstract using the PLOS Medicine headings (Background, Methods and Findings, Conclusions). Please combine the Methods and Findings sections into one section, “Methods and findings”.**

We implemented these changes.

4. **Abstract Background: Provide the context of why the study is important. The final sentence should clearly state the study question. Some of the content in the Introduction should be relocated to the Methods and Findings (e.g., outcomes, length of follow up).**

We implemented these changes. The new Background Paragraph in the Abstract now reads:

*“****Background:*** *Hypertension represents one of the major risk factors for cardiovascular morbidity and mortality globally. Early detection and treatment of this condition is vital to prevent complications. However, hypertension often goes undetected, and even if detected, not every patient receives adequate treatment. Identifying simple and effective interventions is therefore crucial to fight this problem and allow more patients to receive the treatment they need. Therefore, we aim at investigating the impact of a population-based blood pressure screening and the subsequent “low-threshold” information treatment on long term cardiovascular disease (CVD) morbidity and mortality.”*

5. **Abstract Methods and Findings:**

- **Please include the population, years during which the study took place, length of follow up, and main outcome measures.**
- **Please quantify the main results (with 95% CIs and p values).**
- **In the last sentence of the Abstract Methods and Findings section, please describe the main limitation(s) of the study's methodology.**

We carried out the changes as requested. Following current trends in many areas of empirical research, throughout the manuscript we have chosen to provide confidence intervals instead of p-values. We have done so because confidence intervals are a more informative measure of precision, and because they are less prone than p-values to be interpreted in an over-simplistic, binary manner (see references below). Thus, if possible, we would like to choose not to add p-values to the abstract.

Greenland, Sander, et al. "Statistical tests, P values, confidence intervals, and power: a guide to misinterpretations*." European journal of epidemiology* 31.4 (2016): 337-350. Available at: <https://www.ncbi.nlm.nih.gov/pmc/articles/PMC4877414/pdf/10654_2016_Article_149.pdf>

Benjamini, Yoav, et al. "ASA President’s task force statement on statistical significance and replicability." *CHANCE* 34.4 (2021): 10-11. Available at:

<https://www.tandfonline.com/doi/pdf/10.1080/09332480.2021.2003631>

6. **At this stage, we ask that you include a short, non-technical Author Summary of your research to make findings accessible to a wide audience that includes both scientists and non-scientists. The Author Summary should immediately follow the Abstract in your revised manuscript. This text is subject to editorial change and should be distinct from the scientific abstract. Please see our author guidelines for more information:** <https://journals.plos.org/plosmedicine/s/revising-your-manuscript#loc-author-summary>

We submitted an Author Summary together with the requested changes.

7. **Citations should be normal script, located within square brackets, and appear before punctuation (e.g., [1]).**

We implemented these changes in the references.

8. **Lines 24-25: Please add ‘to our knowledge’, or similar.**

We implemented this change. We also included a similar statement in the Conclusions, as suggested by reviewer 2.

9. **Please label and describe items in your Supporting Information as outlined here:** <https://journals.plos.org/plosmedicine/s/supporting-information>

We have now labelled and structured the Supporting Information as outlined in the guidance.

10. **Please define the abbreviations in Table S1.**

We implemented this change. The full abbreviation list with explanation is:

*“BW=bandwidth, CVD=cardiovascular disease, BMI=body mass index, BP=blood pressure, Med.=Medication, MI=myocardial infarction; mm HG=millimeters mercury”*

11. **Please define "lost to follow-up" as used in this study. Other reasons for exclusion should be defined.**

Thank you for pointing out this unclarity. Similar comments on sample construction and exclusion criteria were also done by reviewer 1, point 7 and reviewer 3, point 1.

In the methods section (page 12), we included a more detailed explanation of what we mean with “loss” or “lost to follow-up”. Furthermore, we provide a more detailed explanation of sample construction in the results section (page 14) with a link to new flowcharts detailing this process (S1 Figure and S2 Figure), as suggested by reviewers 2 and 3.

12. **Did your study have a prospective protocol or analysis plan? Please state this (either way) early in the Methods section.**

**a) If a prospective analysis plan (from your funding proposal, IRB or other ethics committee submission, study protocol, or other planning document written before analyzing the data) was used in designing the study, please include the relevant prospectively written document with your revised manuscript as a Supporting Information file to be published alongside your study, and cite it in the Methods section. A legend for this file should be included at the end of your manuscript.**

**b) If no such document exists, please make sure that the Methods section transparently describes when analyses were planned, and when/why any data-driven changes to analyses took place.**

**c) In either case, changes in the analysis-- including those made in response to peer review comments-- should be identified as such in the Methods section of the paper, with rationale.**

Thank you for pointing this out, we agree with the reviewer that protocols and study plans are essential to ensure transparency and avoid data-driven decisions during the analysis stage. Our group recently committed to publish all protocols and analysis plans upfront, on the Open Science Framework. However, since this analysis was started before this commitment, we regret that we did not apply the same standardized approach and we did not write a protocol and analysis plan. We state this in the manuscript, together with meaningful decisions that were taken and that resulted in substantial changes of our initial analysis plan (page 13, or see below). We guarantee that none of the decisions that were taken while analyzing the data resulted from a previous consultation of the results, but were only taken to increase methodological quality and clinical relevance of the results.

We included the following statement in the paper under the section “Protocol and analysis plan availability” (page 12):

*“We did not develop an a priori study protocol for this study. We initially planned and conducted explorative analyses to determine the feasibility of employing a RD design to evaluate the population-based blood pressure screening on a CVD events. In these analyses we employed a fuzzy RD design using both systolic and diastolic blood pressure as separate cut-offs. After determining that the evaluation was indeed feasible, we set out to conduct this full study. During planning consultations among the team at this stage, we made two main changes to our initial explorative analyses. Firstly, we decided to combine the two cut-offs into a single threshold, the blood pressure score, and to consequently employ a sharp RD design, as described above. Secondly, to increase clinical relevance, we decided not only to assess the composite outcome of any CVD event as a primary outcome, but to also assess any fatal CVD event. During the study we ensured that we made decisions based only on methodological considerations and that no decisions were based on the results of the analyses.”*

13. **Thank you for including a completed TREND checklist. Please use section and paragraph numbers rather than page numbers when completing the checklist and cite the checklist in your Methods section (as S1 checklist, or similar).**

As requested, we have now adapted the TREND checklist by including the section and paragraph number for each respective item. The checklist is now linked in the text (page 13) and is called “S1 checklist”.

14. **Please remove the ‘Disclosures’ section from the end of the main text and relocate the content regarding ethical approval and consent to the Methods section. The Data Availability information can be removed as this is captured via your Data Availability Statement in the submission form.**

We implemented these changes. The content regarding ethical approval and consent was relocated to page 6 (Methods section).

15. **Similarly, the Sources of funding section can be removed and the information instead entered in the Financial Disclosure section of the submission form.**

We implemented these changes.

16. **Figure S1: Please update ‘Gender’ to ‘Sex’.**

We have carried out this change. Please note that we changed the numbering of the supplementary figures as suggested, old Figure S1 is now S4 Figure.

17. **Line 396: Please temper claims to primacy by adding ‘to our knowledge’, or similar.**

We implemented this change. See also editor´s comment nr. 8 and reviewer 2, point 8.

**Comments from the reviewers:**

**Reviewer #1:**

**This study evaluates the effect of blood pressure screening, in the context of a cohort study, on outcomes of blood pressure and fatal or nonfatal CVD events over some 16 years of follow-up. The study addresses a well-known issue and provides new information to inform ongoing debate. The data are analysed using sophisticated methods and the paper is well organised.**

1. **It is not clear what is the comparator. This is a single group study, but it appears that this intervention did not improve on existing blood pressure care. The paper needs to specify what the latter is. In the UK, general practitioners are encouraged to record the blood pressure of their patients every few years, and a recent cardiovascular health check programme has been found to provide limited additional benefit. Does a similar arrangement exist in Germany?**

We thank the reviewer for raising this issue. In order to make sure that the intervention and comparator are clear, we have adapted the text at multiple points in the text. We explain what intervention and comparator are at the beginning of the paragraph “Study design and statistical analysis” (pages 8-9). We now clearly mark intervention and control group in the text:

*“We evaluated the impact of a hypertension diagnosis and encouragement letter on health outcomes using a RD design. RD enables causal inference when an intervention is assigned based on a threshold rule. We compared KORA study participants who had baseline blood pressure readings just below the threshold for hypertension (and therefore did not receive an information treatment comprising hypertension diagnosis and encouragement to seek care) (control group) with study participants who had baseline blood pressure readings just above the threshold (and therefore received hypertension diagnosis and an encouragement prompt) (intervention group).”*

Since we are analyzing an intervention outside of the healthcare sector (see new explanation on page 7, reviewer 2 comment 2), we are basically comparing individuals who received the intervention within the epidemiological study, with individuals who did not receive the intervention within the same study. As we state in the same paragraph reported above, any factor outside of the analyzed study is treated as randomized between the two groups on both sides in a vicinity of the cutoff (i.e. intervention and control group). This means that we assume that both groups had access to the same routine healthcare, so that this factor does not impact the validity of our results. This is one of the main advantages of RD designs. We included the following sentences following the paragraph above (page 9):

*“Due to random variability in blood pressure measurements, participants just above / below the threshold, apart from receiving the information treatment, can be assumed to be similar on both observed and unobserved baseline characteristics, as in a randomized trial. This means that differences outside of the study context, such as differing access and standards of care, should also be balanced across intervention and control groups.”*

2. **Use of the term 'screening' can lead to misunderstanding. While public health workers refer to 'population screening', general practitioners and other clinicians use the term 'screening' when they routinely perform tests on specific groups of patients. It would be unfortunate if readers of this paper took from it the message that recording BP is not helpful.**

We agree with the reviewer that this is an important point. In the abstract and in the Methods section we now explicitly state that we analyze a “screening” that is embedded within a population-based epidemiological study. This should clarify any doubts about the setting in which the screening was performed. Concretely, the part that we added in Methods section (page 7) now reads:

*“For this study, we focus on communication of the results of the blood pressure (BP) screening, which we consider a “light touch” intervention since individuals were simply informed of their diagnosis and encouraged to consult a physician through their own initiative.*

*As such, the assessed intervention is an information treatment targeted at high-risk individuals, following a blood pressure screening within a population-based epidemiological study. The intervention represents a one-time occurrence outside of the usual healthcare context, and is therefore different than regular screening interventions carried out as part of primary care.”*

3. **The results are generally negative. However, the confidence intervals for the main estimates are very wide leading to questions concerning whether the power of the study is sufficient. Further, 'absence of evidence is not evidence of absence' so it may not be justified to conclude that screening 'did not reduce long-term fatal or nonfatal CVD events' - there is insufficient evidence to draw a conclusion.** <https://www.bmj.com/content/311/7003/485>

We thank reviewer 1 and 2 for pointing out this issue (reviewer 2, comments 3 and 10; reviewer 1, comment 6). We answer to these commentaries here.

Throughout the manuscript, we changed the way that we present this null-finding, for example by stating that the analysis “does not suggest an effect of information intervention”. See relevant points in the abstract, results (pg. 15), discussion (pg. 16-17) and conclusion (pg. 22). In the discussion, we specifically included a more detailed explanation of this issues as potential interpretation for our results:

*“…Second, the effect of this “light touch” intervention was not sustainable or large enough to translate into an identifiable effect on CVD morbidity or mortality, within our data. RD design require in fact a large number of observation within the bandwidth around the cutoff, so it is possible that our analysis was not sufficiently powered to detect small changes.”*

4. **In the Abstract where it concludes 'More intensive interventions, along with nudging and/or education, may be necessary to affect individual behavior and long-term outcomes.'. This needs to be set in the context of services that already exist (as noted above). No evidence is presented on nudging or education so these ideas need not be mentioned in the conclusion.**

Thank you for pointing this out. We changed the Conclusion section of the abstract accordingly:

*“The study does not provide evidence of an effect of the assessed blood pressure screening and subsequent information treatment on blood pressure and behavior, but also on long-term CVD mortality and morbidity. Future studies should consider larger datasets to detect possible effects and a shorter follow-up for the intermediate outcomes (i.e. blood pressure and behavior) to detect short-, medium- and long-term effects of the intervention along the causal pathway.”*

5. **In the Introduction, this is generally good, but it needs to consider the medical care context as mentioned above. What access did participants have to routine BP care?**

Thank you for raising this point. We discuss the role of routine care in reviewer 2, point 2. To make this point clearer, we include in the introduction the following specification:

“We hypothesized that the information given after a blood pressure screening would have motivated patients to seek medical attention. *In the context of routine care in Germany this would likely have led to initiation of a series of further diagnostic*, behavioral and therapeutic measures to keep blood pressure levels under control and to reduce long-term CVD morbidity and mortality.”

6. **The Methods are described clearly and in detail. However, at line 127 the term 'bandwidth' is introduced without explanation and the meaning may not be clear. Later it refers to 'optimal bandwidth', with the meaning also unclear.**

We included an explanation of bandwidth and optimal bandwidth (with references) in the paragraph “Study design and statistical analysis” (pages 8-9). This should help the reader to understand the subsequent methods more clearly:

*“In the analysis, individuals who are just above / below the cutoff are selected using a bandwidth, which determines the span of the distribution around the cutoff in terms of the assignment variable to be considered in the analysis (e.g. a bandwidth of 5mmHg would imply considering individuals between 135 mmHg and 145 mmHg for systolic blood pressure). The optimal bandwidth is computed by optimizing the tradeoff between being in the closest proximity of the cutoff to improve comparability of observations, while at the same time ensuring that enough observations are considered in the analysis.”*

7. **A flowchart detailing the sample selection would be helpful. Where it reads 'The follow-up data for secondary outcomes included 5,130 individuals', please explain why this is reduced as compared to the full sample.**

We provide a flowchart (S1 and S2 Figures), that is mentioned in the Results section. This issue was raised also by reviewer 3, point 1.

The analysis for the secondary outcomes included less individuals than the main analysis because for two of the original KORA studies considered (S1 and S2) no follow-up was available. For the other two studies (S3 and S4) comparable follow-ups were available, surveying the analyzed secondary/intermediate outcomes after 7 years (on average). This is explained in the paragraph “Outcomes” in the methods section (pages 7-8). Furthermore, we included a more detailed overview of sample construction and exclusion criteria in the Results section (page 14). The new flowcharts in the appendix are linked here (page 14).

We also spotted a small mistake in reporting the final sample size of the secondary outcomes sample: the final sample consists of 5,085 individuals, not 5,084 as previously reported. We corrected this in the manuscript.

8. **Where it says 'The graphical inspection of these plots confirmed the absence of manipulation around the respective cutoffs.' please explain what is meant by manipulation and why the graphs demonstrate this.**

We included an explanation of this issue in the manuscript (page 14):

“The graphical inspection of these plots confirmed the absence of manipulation around the respective cutoffs, *since the density curve runs smoothly through the cutoff (if a manipulation had occurred, we would have seen a jump in the distribution of these variables close to the cutoff).”*

9. **The Figure legends could be more informative. For example, Figure 2 appears to show more than the effect at threshold. Figure 3, meaning of the x axis label of 'bandwidth' may not be clear.**

We agree with the reviewer that the figures are every informative, for example they show the relationship between the running variable (blood pressure score) and the outcome (CVD mortality and morbidity) before and after the cutoff. By keeping the explanation simple, we wanted to focus the attention of the reader to the most important result from the figure (namely the jump at the cutoff) rather than mentioning further details that are not relevant towards understanding the final results. We now provide a more detailed figure caption as follows:

“*Notes: graphical representation of the effect at the threshold, with a linear association between the assignment variable (blood pressures score) and the respective outcomes for individuals just below and just above the intervention cutoff (blood pressure score = 0) for (a) 16.9 years fatal CVD event and (b) 16.9 years any CVD event.”*

Thank you for pointing out that the label “bandwidth” might not be clear. As suggested in reviewer 1, point 6, we now introduce in the text an extensive explanation of what “bandwidth” means, in order to familiarize the reader with this concept early in the manuscript. We also add an explanation to the caption of figure 3. The whole caption now reads:

“*Notes: graphical representation of sensitivity analysis results on the effect of the intervention at the threshold (and respective 95% confidence intervals) by changing the bandwidth around the cutoff for (a) fatal CVD events results and (b) any CVD event. The dotted line indicates the result with the optimal bandwidth from the main analysis. Other bandwidths indicate larger or shorter bandwidths: a larger bandwidth indicates that we are considering individuals farther away from the cutoff but also a larger number of observations (i.e. up to 1.4 units on the constructed BPS score around the cutoff). A shorter bandwidth indicates that we are considering individual closer to the cutoff and therefore less observations (i.e. up to 0.2 units on the constructed BPS score around the cutoff).”*

10. **In the Discussion, where it says ' The analysis indicated no effect of a population-based blood pressure screening intervention on long-term CVD mortality and morbidity in Germany.' It really means 'no additional effect' over and above existing BP care. But see also point 3 above.**

Thank you for raising this issue. Please see answer to reviewer 1, point 3.

11. **The remainder of the discussion and referencing is generally good. It may be worth referring to the distinction of population-wide versus high risk approaches from G Rose, strategy of preventive medicine. It may also be useful to refer to behavioural science literature as finding that ‘simply informing patients of their hypertension status is not enough’ may not be surprising.**

Thank you for these suggestions. We now explained our intervention in more detail in the Methods section (page 7), also referring to Rose´s concepts and specifying that *“the assessed intervention is an information treatment targeted at high-risk individuals, following a blood pressure screening within a population-based epidemiological study”.*

Furthermore, we specifically refer to the insights from behavioral sciences in our conclusion, as suggested.

**Reviewer #2:**

**Points of detail**

1. **Page 1: I wonder whether the title should include the location? I suggest Germany rather than Bavaria or Augsburg.**

We implemented this change. See Editor´s comment nr. 1.

2. **Page 6 and elsewhere: The phrase ‘information treatment’ does not seem quite right to me as it made me think that it was the information which was being treated. How about ‘information intervention’?**

Based on several reviewer and editor comments we have now aimed to ensure that the intervention we assessed is very clear and understandable to the reader (see response to reviewer 1, point 2). Thus, we feel now we are quite explicit about what we mean by ‘information treatment’. Additionally, this is a term that is used for interventions of this nature in the literature, including in other papers published by PLOS journals (see links below). If possible, we would prefer to continue using ‘information treatment’ for our study.

<https://journals.plos.org/plosone/article?id=10.1371/journal.pone.0249355>

<https://journals.plos.org/plosone/article?id=10.1371/journal.pone.0259059>

<https://journals.plos.org/plosone/article?id=10.1371/journal.pone.0151469>

3. **Page 7: I think including the question in the original language is a good idea since the authors’ gloss clarifies an important point that diagnosed here did not mean by a registered medical practitioner which I suspect many Anglophones would have assumed.**

We agree with the reviewer that this is an important point. The original question and a translation thereof is included in the footnotes of page 8.

4. **Page 9: Using µ for the error seems unusual, I would have expected ε. For completeness defining βi would be good.**

Thank you for pointing this out. We now express the error term as epsilon and describe the different βi included.

5. **Page 12: It might be best to make clear how the density plots demonstrate the absence of manipulation**

We included an explanation of this issue in the manuscript (page 15):

“The graphical inspection of these plots confirmed the absence of manipulation around the respective cutoffs, *since the density curve runs smoothly through the cutoff (if a manipulation had occurred, we would have seen a jump in the distribution of these variables close to the cutoff).”*

6. **Page 14 I think strictly speaking the analysis failed to find an effect rather than indicating no effect. A similar phrase is found on page 13.**

Thank you for pointing this out. We respond to this important comment under reviewer 2, point 1.

7. **Page 18 Although the regression discontinuity design cannot be used to examine what happens to people with much higher blood pressures it seems a shame not to know anything about them. Perhaps this is better treated in a separate paper.**

We agree with the reviewer that the group of individuals with higher blood pressure levels (~95/160 mmHg) represents a very interesting group, especially considering the historical context in which the intervention was carried out and the data collected. As we mention at page 20, an analysis using the higher cutoff would have been technically feasible, but probably including a large bandwidth around the cutoff and with a very low power, since the density of observations around that cutoff is very small. However, we agree that investigating this group of individuals too would have been very interesting and should be object of further considerations.

8. **Page 20 Claiming to be the first is rather bold. First to our knowledge is safer.**

We implemented this change. The new phrase now reads: “*To the best of our knowledge, this is the first study investigating medium and long-term impacts of a population-based screening on morbidity and mortality outcomes.”* (page 23)

Furthermore, we included a similar statement in the Introduction as suggested by the editors (points nr. 8 and nr. 17).

**Points of more substance**

9. **The authors have apparently truncated problematic times in their imputation. I cannot pretend to be the world’s leading expert on mice but I think the facility to do passive imputation would have solved the problem of the impossible times in a more principled way. One of the package vignettes appears to have an example of imposing a similar constraint. These are hard to find, they are linked from page 78 in the manual on CRAN (mice version 3.14.0). It may be that it is in fact impossible to do as I suggest and it is, of course, reassuring that the complete cases analysis is not substantively different. Incidentally I think providing a citation for mice would be a nice idea. Package authors are working for free and this may be the only credit they get. (Full disclosure: I am a CRAN author although I have no connection with mice.)**

We agree with the reviewer that it would be more elegant to tackle the problematic times within the imputation. Therefore, we went through the document provided to check whether the described methods are applicable to our case/problem. Unfortunately, our case is more complicated than the examples described in the document. We would need to apply post-processing combined with some passive imputation since we would need to use a subject specific upper boundary in the squeeze() function. As a consequence, we were not able to adapt the provided code examples to our problem. We also did some further online research (Stack Exchange etc.), but could not find a solution for our problem there either. Furthermore, from what we understood from the provided document and the information retrieved online, we think that the resulting dataset would probably not be substantially different from the one we get with our “manual” approach. And even if there would be some difference, it would be hard for us to judge which of the datasets is "better". Anyway, we are confident that both approaches would lead to very similar results.

We agree with the reviewer that providing a citation for the mice package is an important recognition of the authors. Therefore, we added the respective citation.

**Reviewer #3:**

**In a longitudinal analysis based on 14,592 German adults from four population-based cohort studies free from cardiovascular disease (CVD) and drug treatment at baseline, the Authors investigated the impact of a blood pressure screening and personalized information treatment on CVD morbidity and mortality over a follow-up time of 16.9 years. They concluded no effect of a blood pressure screening intervention on long-term CVD mortality and morbidity and on the medium-term health behaviour or blood pressure intervention. This is a very well written and interesting paper. Statistical approach appears elegant. The discussion and conclusions are well balanced.**

1. **In the Methods Section, a flow chart of the study design would be of interest.**

Thank you for pointing this out. We respond to this commentary under reviewer 1, point 7.

2. **Methods should be improved with more details on information treatment and on the definition of "light touch" intervention. As it is, is it only a mail communication to the patient without direct information to the General Practitioner? If yes, please added a specific statement with explanation (i.e. privacy policy in Germany ecc…).**

We now extended the explanation of our intervention (Methods, paragraph “Intervention”, page 7):

*“For this study, we focus on communication of the results of the blood pressure (BP) screening, which we consider a “light touch” intervention since individuals were simply informed of their diagnosis and encouraged to consult a physician through their own initiative.*

*As such, the assessed intervention is an information treatment targeted at high-risk individuals, following a blood pressure screening within a population-based epidemiological study. The intervention represents a one-time occurrence outside of the usual healthcare context, and is therefore different than regular screening interventions carried out as part of primary care.”*

Furthermore, we discuss in the Discussion section the fact that the intervention is embedded in an epidemiological study, whose primary aim was not conducting the intervention, with a citation to the relevant study suggested (see next point).

3. **Other studies have found a positive association between correct information about BP to GPs and impact on CVD mortality. Any further direct contact between the medical staff involved in the cohort studies and the participants? Other studies have investigated in longitudinal population-based studies the beneficial impact of observational epidemiological studies on the CVD mortality. On the other hand, some studies have outline that long-term negative psychological effects, as a consequence of the label of HT, exist. Therefore, clinicians might also consider appropriate positive health messages in participants with hypertension (doi:10.1136/openhrt-2015-000341). References should be implemented.**

We agree with the reviewer that this is an important point to be considered. We include a reference to this issue in the discussion section (page 22), citing the suggested paper:

“Further research should investigate how different communication and education strategies might impact awareness and motivation to seek further treatment or adhere to recommendations, *as well as potential unintended consequences if, for example, a diagnosis leads to psychological burden (Tikhonoff et al., 2016)”*.
